# Identification and Characterization of the ERF Subfamily B3 Group Revealed *GhERF13.12* Improves Salt Tolerance in Upland Cotton

**DOI:** 10.3389/fpls.2021.705883

**Published:** 2021-08-09

**Authors:** Lili Lu, Ghulam Qanmber, Jie Li, Mengli Pu, Guoquan Chen, Shengdong Li, Le Liu, Wenqiang Qin, Shuya Ma, Ye Wang, Quanjia Chen, Zhao Liu

**Affiliations:** ^1^Engineering Research Centre of Cotton, Ministry of Education, Xinjiang Agricultural University, Urumqi, China; ^2^State Key Laboratory of Cotton Biology, Institute of Cotton Research, Chinese Academy of Agricultural Sciences, Anyang, China; ^3^State Key Laboratory of Cotton Biology, Zhengzhou Research Base, Zhengzhou University, Zhengzhou, China

**Keywords:** ERF, phylogenetic analysis, gene duplication, collinearity analysis, ectopic expression, salt stress, gene silencing

## Abstract

The APETALA2 (AP2)/ethylene response factor plays vital functions in response to environmental stimulus. The ethylene response factor (ERF) subfamily B3 group belongs to the AP2/ERF superfamily and contains a single AP2/ERF domain. Phylogenetic analysis of the ERF subfamily B3 group genes from *Arabdiposis thaliana, Gossypium arboreum, Gossypium hirsutum*, and *Gossypium raimondii* made it possible to divide them into three groups and showed that the ERF subfamily B3 group genes are conserved in cotton. Collinearity analysis identified172 orthologous/paralogous gene pairs between *G. arboreum* and *G. hirsutum*; 178 between *G. hirsutum* and *G. raimondii*; and 1,392 in *G. hirsutum*. The *GhERF* subfamily B3 group gene family experienced massive gene family expansion through either segmental or whole genome duplication events, with most genes showing signature compatible with the action of purifying selection during evolution. Most *G. hirsutum* ERF subfamily B3 group genes are responsive to salt stress. *GhERF13.12* transgenic Arabidopsis showed enhanced salt stress tolerance and exhibited regulation of related biochemical parameters and enhanced expression of genes participating in ABA signaling, proline biosynthesis, and ROS scavenging. In addition, the silencing of the *GhERF13.12* gene leads to increased sensitivity to salt stress in cotton. These results indicate that the ERF subfamily B3 group had remained conserved during evolution and that *GhERF13.12* induces salt stress tolerance in Arabidopsis and cotton.

## Introduction

Plants are sessile organisms continuously challenged by changing environmental conditions. As a result, different signal cascades mediate plant metabolism and development to ensure adaptation to these environmental challenges (Markakis et al., 2012). Transcription factors (TFs) are key regulators that play important roles in various stress responses (Debbarma et al., 2019). In Arabidopsis, the plant specific TF APETALA2/ethylene-responsive element binding factor (AP2/ERF) constitutes one of the largest among 65 TF families (Nakano et al., 2006; Mitsuda and Ohme-Takagi, 2009). AP2 TF was first isolated from Arabidopsis in 1994 (Jofuku et al., 1994). Since then, the total number of identified plant AP2/ERF increased from 147 in *Arabidopsis thaliana* to 210 in *Zea mays* (Liu et al., 2013; Guo et al., 2016). The AP2/ERF family is a nuclear protein with 68 amino acid repeat motifs and possesses DNA-binding activity (Ohme-Takagi and Shinshi, 1995). Based on its evolutionary structure, AP2/ERF was divided into three distinct subgroups (Riechmann et al., 2000). The AP2 subfamily members (14 in Arabidopsis) contain double AP2/ERF domains, the RAV subfamily (six members) contains a single AP2/ERF domain and a B3-DNA binding domain, while another subfamily (125 members) contains only one AP2/ERF domain (Sakuma et al., 2002). Based on phylogenetic and similarity analyses of the AP2/ERF domain, these 125 members were divided into a DREB subfamily (group A; 56 members), an ERF subfamily (group B; 65 members), and others (four members). The DREB and ERF subfamilies were further subdivided into six groups, from A1–A6 and B1–B6, respectively. The ERF subfamily B3 group contains a single AP2 domain with β-1, β-2, β-3, and α-helix regions (Heyman et al., 2018; Cao et al., 2020). To date, the ERF TF family has been identified in Arabidopsis (Nakano et al., 2006), rice (Rashid et al., 2012), cotton (Jin and Liu, 2008), maize (Liu et al., 2013), soybean (Zhang et al., 2008), wheat (Zhuang et al., 2011), sorghum (Yan et al., 2013), and various other species.

Previous reports identified the roles played by ERF TFs in regulating biotic and abiotic stress responses by repressing or activating abscisic acid (ABA)-related gene expressions (Licausi et al., 2013; Mizoi et al., 2013). For example, the overexpression of *AtERF4* inhibits root elongation and negatively regulates signaling of ABA and ethylene (Yang et al., 2005). *AtERF7* represses ABA-responsive genes (Zhang et al., 2007), and *ERF111* negatively regulates ABA responses and stress-related gene expression (Pandey et al., 2005). Similarly, *AtERF13* belongs to the B3 group of the ERF subfamily, and the overexpression of *AtERF13* leads to hypersensitivity to glucose, whose effect is mediated by ABA (Lee et al., 2010). *AtERF15* positively regulates ABA responses in Arabidopsis (Lee et al., 2015), and *BpERF13* positively responds and regulates cold-induced stress response (Lv et al., 2020). The overexpression of *ERF1-V* from *Haynaldia villosa* increases tolerance to salt stresses in wheat (Xing et al., 2017). Similarly, another wheat ERF gene, *TaERF3*, promotes tolerance to salt stresses. Moreover, the amount of proline and chlorophyll in *TaERF3*-overexpressing wheat treated with 250 mM NaCl was higher than that of control plants, while the amount of hydrogen peroxide showed an inverse pattern (Rong et al., 2014). *CitERF13* is involved in the regulation of photosynthesis by transient overexpression in *Nicotiana tabacum* leaves (Xie et al., 2017). Further, *ERF95* (or *ESE1, Ethylene and Salt Inducible 1*) and *ERF98* regulate salt tolerance in Arabidopsis (Zhang L. et al., 2011; Zhang et al., 2012), while *ERF97* (previously named *AtERF14*) mediates defense response in Arabidopsis (Catinot et al., 2015).

Cotton is a major source of natural fibers for the textile industry and an excellent model to study polyploidy (Malik et al., 2018; Yang et al., 2020). Cotton plants are grown in over 70 countries worldwide and play an important role in the global economy (Qiao et al., 2008). However, various abiotic stresses, such as drought and salt, affect cotton plant growth and yield (Wang et al., 2017; Chen et al., 2019; Qanmber et al., 2019b). Moreover, it has been shown that soil salinity ranging from 8–18 dS/m results in 10–55% yield loss in cotton (Satir and Berberoglu, 2016). Therefore, abiotic stresses, such as salinity, constitute a serious problem that merits further in-depth study. Transcriptomic data analysis identified 2,356 differentially expressed genes between two cotton samples (saline or water-treated), in particular among transcription factors that were classified as ERF members (17.6%). Furthermore, when compared with the control, cotton seedlings silenced with GhERF4L and GhERF54L were significantly less tolerant to salt stress (Long et al., 2019). Finally, previous studies found that *GhERF1, GhERF 2, GhERF 3, GhERF6*, and *GhERF38* mediate salt and drought tolerance (Qiao et al., 2008; Jin et al., 2010; Ma et al., 2017).

In this study, we identified the ERF subfamily B3 group in three cotton species, namely *G. arboreum, G. hirsutum*, and *G. raimondii*. In order to study the evolution of the ERF subfamily B3 group, we studied the genes, phylogeny, sequence logos, duplication events, collinearity, selection pressure (*Ka/Ks* non-synonymous/synonymous), and chromosomal location. In addition, we determined the expression patterns of genes with salt stress in specific tissues. Subcellular localization analysis was performed for *GhERF13.12*. We overexpressed *GhERF13.12* in Arabidopsis with ACC (1-aminocyclopropane-1-carboxylic acid) treatment and subsequently studied the effects of salt stress by analyzing POD activity, the content of DAB, MDA, H2O2, total chlorophyll, and soluble sugars, as well as salt-responsive gene expression. We then evaluated the role played by *GhERF13.12* in salt tolerance in cotton by silencing the gene. Collectively, this study provides an understanding of the ERF subfamily B3 group and the role played by *GhERF13.12* in cotton salt tolerance.

## Materials and Methods

### Sequence Identification and Retrieval

We identified the members of the ERF subfamily B3 group gene family in Arabidopsis by keyword searching in the Arabidopsis database (http://www.arabidopsis.org) and downloading the corresponding protein sequences from TAIR 10 (http://www.arabidopsis.org). These protein sequences were then used as query in Local BLASTP to identify the ERF subfamily B3 group genes in *G. arboretum* (Institute of Cotton Research, ICR, version 1.0) (Du et al., 2018), *G. hirsutum* (Nanjing Agricultural University, NAU, version 1.1) (Zhang et al., 2015), and *G. raimondii* (Joint Genome Institute, JGI, version 2.0) (Paterson et al., 2012). We downloaded the databases for different plant species, such as, *G. raimondii, G. hirsutum*, and *G. arboreum*, from the Cotton Genome Database (https://www.cottongen.org/) with an e-value cutoff of 1e−5 (Yu et al., 2014). Moreover, we obtained ERF subfamily B3 group gene pfam ID (PF00847) from the Pfam website (http://pfam.janelia.org/) (Finn et al., 2016) and further verified the identified ERF subfamily B3 group gene family members in *G. arboreum, G. hirsutum*, and *G. raimondii* using a hidden Markov model (HMM).

### Phylogenetic and Sequence Logos Analysis

To understand the evolutionary relationship of ERF subfamily B3 group gene family members, full-length protein sequences of *A. thaliana, G. arboreum, G. hirsutum*, and *G. raimondii* were aligned in Clustal X1.81 (http://www.clustal.org/), and an evolutionary tree was constructed using the NJ method in MEGA 7.0 (Kumar et al., 2016), as described previously (Qanmber et al., 2018; Wang et al., 2018; Yu et al., 2018). To estimate the reliability of clade classification, we selected 1,000 replicates using the bootstrap method. A Poisson model with default parameters enabled us to estimate the substitution rate.

### Collinearity, Ka/Ks Values, and Chromosomal Location

To perform collinearity analysis, orthologous and paralogous gene pairs were obtained as previously described (Qanmber et al., 2019b,c; Ali et al., 2020). Shortly, orthologous and paralogous gene pairs belonging to the cotton ERF subfamily B3 group genes were retrieved by an all-vs. all search in BLASTP. The results were further subjected to MCScan analysis to generate collinear blocks within and between cotton A, D, At, and Dt genomes and sub-genomes. We then used the MCScanX software to estimate collinear pairs and gene duplication event types in the cotton ERF subfamily B3 group genes, and the CIRCOS software was used to draw the final figure (Krzywinski et al., 2009; Zheng et al., 2020). Further, in order to calculate the *Ka/Ks* values, we aligned the protein sequences of the ERF subfamily B3 group genes using Clustal X1.81 (http://www.clustal.org/) and converted the protein sequences of the ERF subfamily B3 group genes into cDNA sequences using the PAL2NAL package (http://www.bork.embl.de/pal2nal/). Finally, we obtained synonymous (*Ks*) and non-synonymous (*Ka*) divergence level values using the “simple *KaKs* calculator” software in TBtools (Chen et al., 2020).

### qRT-PCR Analysis

For qRT-PCR analysis, we extracted RNA using the RNAprep Pure Plant Kit (TIANGEN, Beijing, China). We assessed the concentration and quality of the RNA by NanoDrop2000 (Thermo Fisher Scientific, Waltham, MA, United States), and then synthesized 1 μg of RNA into cDNA with TransScript® RT All-in-One First-Strand cDNA Synthesis SuperMix for qPCR (One-Step gDNA Removal) (https://www.transgen.com.cn/news_show/927.html) as previously described (Xiong et al., 2019). The following parameters were used for the RT-PCR procedure: 94°C for 3 min, 28 cycles of 10 s at 98°C, 10 s at 59°C, and 10 s at 72°C. The 2 × T5 Super PCR Mix (Colony) was used. The PCR products were detected by electrophoresis in a 1.5% agarose gel. *Actin2* (*AT3G18780.1*) and *GhHis3* (*AF024716*) were used as internal controls, and SYBR Green was used to conduct the qRT-PCR analysis on a LightCycler 480 (Roche Diagnostics GmbH, Mannheim, Germany). Each experiment was repeated three times (biological repeats), and the relative gene expression values were calculated using the 2^−ΔΔCT^ method (Livak and Schmittgen, 2001). The primers used in this experiment are listed in Supplementary Table 5. Values are shown as the mean ± SE (*n* = 3, Student *t*-test, ^*^P < 0.05, ^**^P < 0.01, and ^***^P < 0.001).

### Vector Construction and Transformation

For subcellular localization, full-length coding sequences of the *GhERF13.12* gene were inserted in a pEG101 vector expressing YFP, and then a vector expressing the GhERF13.12-YFP fusion protein was constructed (Zhao et al., 2019). The construct in GV3101 was then transformed into 2-cm diameter leaves of 1-month-old tobacco (*N. benthamiana*) cells for subcellular localization analysis. The plants were placed in the dark for 24 h, and tobacco epidermal cells were observed after 72 h of inoculation using a fluorescence microscope.

For the ectopic expression of the *GhERF13.12* gene in Arabidopsis, full-length cDNA sequences were amplified by PCR using gene-specific primers, cloned into a pCAMBIA-2300 vector with a constitutive Cauliflower mosaic virus 35S promoter, and then transformed into Arabidopsis *via* the floral dip method using the *Agrobacterium tumefaciens* strain GV3101 (Zhang et al., 2006). For the silencing of the *GhERF13.12* gene in cotton, we cloned a 300-bp fragment of the coding sequence (CDS) with high specificity into a pTRV-RNA2 vector using *Xba*I and *Sma*I restriction sites to generate TRV::*GhERF13.12*. Similarly, TRV::*GhCLA1* was constructed as a visual marker for monitoring silencing efficiency. TRV::00 (empty vector) was used as the negative control. We transformed these constructed vectors into the Agrobacterium tumefaciens strain GV3101.

### Plant Material and Treatments

We used the wild-type (WT) Columbia-0 (Col-0) ecotype for ectopic expression and subsequent analysis in Arabidopsis. For cotton, the *G. hirsutum* ZM24 variety was used to collect the samples used for tissue specific expression. Cotton plants at three distinct leaf stages were treated with 400 mM NaCl for 6, 12, and 24 h using the ZM24 variety for salt treatment and virus-induced gene silencing (VIGS) (Qanmber et al., 2019b). The cotton plant ZM9807 (salt-tolerant) was used as a positive control. Furthermore, 250 mM NaCl was used to stress Arabidopsis seedlings for 9 days. We then collected these samples, which were immediately frozen in liquid nitrogen and stored at −80°C until they were used for RNA extraction. Arabidopsis plants were grown under conditions of 22°C on a 16 h light/8 h dark photoperiod. WT and *GhERF13.12* transgenic Arabidopsis seedlings were incubated in a MS medium with or without 10 μM ACC for 3 days in darkness in order to check the ethylene-evoked triple response. Cotton seeds were planted in vegetative soil and vermiculite (v/v = 1:1) in flower pots and grown at 30°C on a 16 h light/8 h dark photoperiod.

### Physiological Analysis

We measured various physiological indices using the Solarbio kit following the instructions of the manufacturer. A UV-1600 spectrophotometer (AOE Instruments, Shanghai, China) was used to measure absorbance (Qanmber et al., 2019b). We harvested 0.2 g fresh leaves of WT and transgenic plants. Malondialdehyde (MDA) content was measured at 450, 532, and 600 nm as previously described (Rao and Sresty, 2000). To evaluate the amount of soluble sugar, 1 ml distilled water was added into 0.2 g of leaf materials; the mixture was ground and homogenized and then boiled for 10 min in a centrifuge tube. The mixture was centrifuged at 8,000 g for 10 min at room temperature, and then the absorbance of the supernatant was measured at 620 nm. Total chlorophyll content was estimated by measuring the absorbance in the extracts at 663 and 645 nm. Peroxidase (POD) was measured as mentioned in a previous study (Wang et al., 2012). Further, 3, 3'-diaminobenzidine (DAB) was used to measure the H_2_O_2_ levels and the staining was stopped until the difference could be observed and decolorized with 75% ethanol as previously described (Zhang X. et al., 2011). Classification of salt damage levels and salt damage index was determined as previously described (Qanmber et al., 2019b). Values are the means ± SE (*n* = 3, Student *t*-test, ^*^P < 0.05, ^**^P < 0.01, and ^***^P < 0.001).

## Results

### Identification of the ERF Subfamily B3 Group Gene Family

A total of 148 members of the ERF subfamily B3 group were identified in four plant species using different *in silico* approaches, such as the SMART (http://smart.embl-heidelberg.de/) (Letunic et al., 2015), PROSITE (http://prosite.expasy.org/), and InterProscan 63.0 (http://www.ebi.ac.uk/interpro/) (Jones et al., 2014) websites, as described in previous studies (Liu et al., 2018; Faiza et al., 2019; Li et al., 2019; Qanmber et al., 2019a). Among these, 18 ERF subfamily B3 group genes were found in *A. thaliana*, 18 in *G. arboretum* (diploid cotton), 94 in *G. hirsutum* (tetraploid cotton), and 18 in *G. raimondii* (diploid cotton) (Supplementary Table 1). The highest number of genes was, thus, found in *G. hirsutum*, indicating that the ERF subfamily B3 group experienced large scale expansions in this species. Notably, *G. hirsutum* had more ERF subfamily B3 group genes than both *G. raimondii* and *G. arboreum*, demonstrating polyploidy and massive gene duplication events in the evolutionary process. As the main object of this study was *G. hirsutum*, we compared the ERF subfamily B3 group genes identified in this species from NAU with JGI, ICR, and HAU genome sequence databases. As we found no differences, we proceeded with the ERF subfamily B3 group genes retrieved from NAU.

### Phylogenetic and Sequence Logos Analysis

**Figure 1:**
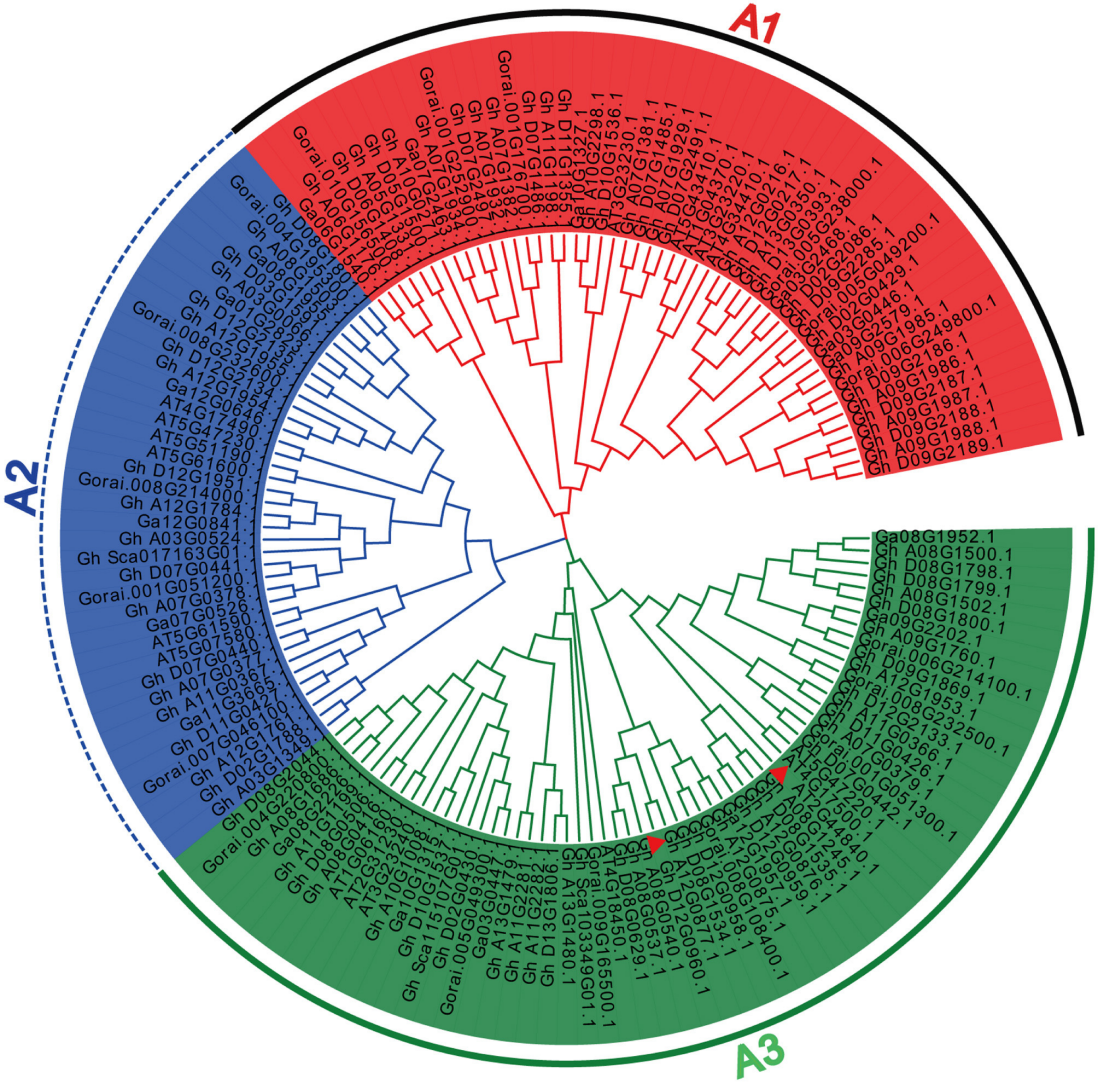
Phylogenetic analysis of the (ERF) subfamily B3 group gene family. The 148 genes were divided into three groups (A1–A3). These genes were found in *A. thaliana, G. arboreum, G. hirsutum*, and *G. raimondii*. The NJ method was used to build the phylogenetic tree, and topological robustness was evaluated by bootstrapping with 1,000 replications. The cotton and Arabidopsis ERF accessions are indicated. The gene marked with a red triangle corresponds to *ERF13* in Arabidopsis and cotton.

To estimate the evolutionary relationship among the ERF subfamily B3 group genes, we constructed a phylogenetic tree of this gene family in four plant species, namely, *A. thaliana, G. arboreum, G. hirsutum*, and *G. raimondii*. Phylogenetic analysis divided all the ERF subfamily B3 group genes into three groups, from A1 to A3 (Figure 1), according to Arabidopsis group classification (Nakano et al., 2006). Group A3 is the largest, containing 60 ERF subfamily B3 group genes. In contrast, group A2 is the smallest, with a total of 38 genes. Group A1 contains a total of 50 ERF subfamily B3 group genes, of which 5 are found in *A. thaliana*, 6 in *G. arboreum*, 33 in *G. hirsutum*, and 6 in *G. raimondii*. Group A2 contains a total of six, six, 21, and five ERF subfamily B3 group genes in *A. thaliana, G. arboreum, G. hirsutum*, and *G. raimondii*, respectively. Similarly, group A3 contains 7, 6, 40, and 7 ERF subfamily B3 group genes in *A. thaliana, G. arboreum, G. hirsutum*, and *G. raimondii*, respectively. Accordingly, all groups represented in the ERF subfamily B3 group are present in all species. Phylogenetic analysis demonstrated that the ERF subfamily B3 group genes found in *G. hirsutum* experienced maximum gene family expansion compared with other species, as the homologous/orthologous pairs clustered together across all three groups. Further, *A. thaliana* and *G. hirsutum* showed gene pairs that originated from the same node, indicating that the *A. thaliana* and, especially, the *G. hirsutum* ERF subfamily B3 group genes experienced massive gene duplication and gene family expansion. This suggests gene duplication was a major reason behind the expansion of the ERF subfamily B3 group gene family. However, it is possible that gene duplication differed between groups and plant species.

To find whether the ERF subfamily B3 group genes were conserved throughout the evolutionary process, we constructed sequence logos of the ERF subfamily B3 group for *G. arboreum, G. raimondii*, and *G. hirsutum* (Supplementary Figure 1). The results predicted a very similar distribution of amino acid residues in the protein sequences of the different species, such as in the N and C terminals. These results indicate the cotton ERF subfamily B3 group genes were highly conserved in the course of evolution.

### Gene Duplication, Collinearity, and Ka/Ks Values Analysis

As the phylogenetic analysis evidenced the occurrence of gene duplication events during cotton evolution, we next explored which types of gene duplication took place in the ERF subfamily B3 group in each species. A total of 15 whole genome duplications (WGDs) and segmental duplications, along with two dispersed duplications, were identified in *G. arboreum*. We also found 13 WGDs and segmental duplications, in addition to a dispersed duplication in *G. raimondii*. Finally, we identified 71 WGDs and segmental duplications, 22 dispersed, and singleton duplication events in *G. hirsutum* (Supplementary Table 2). These results demonstrate that WGDs and segmental duplications were the major duplication type throughout the evolution of the ERF subfamily B3 group gene family in cotton.

**Figure 2:**
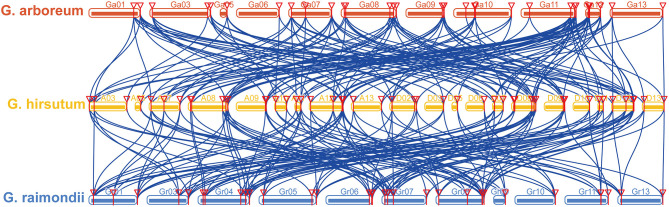
Multiple collinearity analysis of cotton ethylene response function (ERF) subfamily B3 group genes. Multiple collinearity analysis found collinearity blocks among *G. arboretum* (red), *G. hirsutum* (yellow), and *G. raimondii* (blue).

Next, we performed collinearity analysis among three observed cotton species, namely, *G. arboreum, G. hirsutum*, and *G. raimondii*. We identified 172 orthologous and paralogous gene pairs between *G. arboreum* and *G. hirsutum*. Among these, 87 were found between the A-subgenomes of *G. hirsutum* and *G. arbortum*, while 85 orthologous/paralogous gene pairs were found between the D-subgenomes of *G. hirsutum* and *G. arboreum*. Similarly, we identified 178 orthologous/paralogous gene pairs between *G. hirsutum* and *G. raimondii*, of which 91 and 87 occurred between the A- and D-subgenomes of *G. hirsutum* and *G. raimondii*, respectively (Figure 2, Supplementary Table 3). Surprisingly, no orthologous/paralogous gene pairs were identified on chromosomes 2 and 4 of *G. arboreum*; chromosomes A01, A02, A04, A06, D01, and D04 of *G. hirsutum*; and chromosomes 2 and 12 of *G. raimondii*. We were also able to identify 1,392 orthologous/paralogous gene pairs in *G. hirsutum* within and between the A- and D-subgenomes (Supplementary Figure 2, Supplementary Table 3).

The *Ka/Ks* (non-synonymous/synonymous) analysis of the orthologous/paralogous gene pairs between *G. arboreum, G. hirsutum*, and *G. raimondii*, as well as within *G. hirsutum*, demonstrated 339 pairs between the three species with *Ka/Ks* < 1, including 315 pairs with *Ka/Ks* < 0–0.5, 24 pairs with *Ka/Ks* < 0.5–1, and 11 pairs with *Ka/Ks* > 1 (Supplementary Table 3). Similarly, all (1,392) the orthologous/paralogous gene pairs within *G. hirsutum* showed *Ka/Ks* < 1 (1,362 pairs with *Ka/Ks* < 0–0.5 and 30 pairs with *Ka/Ks* < 0.5–1) (Supplementary Table 4). We hypothesize that most orthologous/paralogous gene pairs in cotton underwent purifying selection in the course of evolution.

### Expression Patterns Analysis and Subcellular Localization of *GhERF13.12*

**Figure 3:**
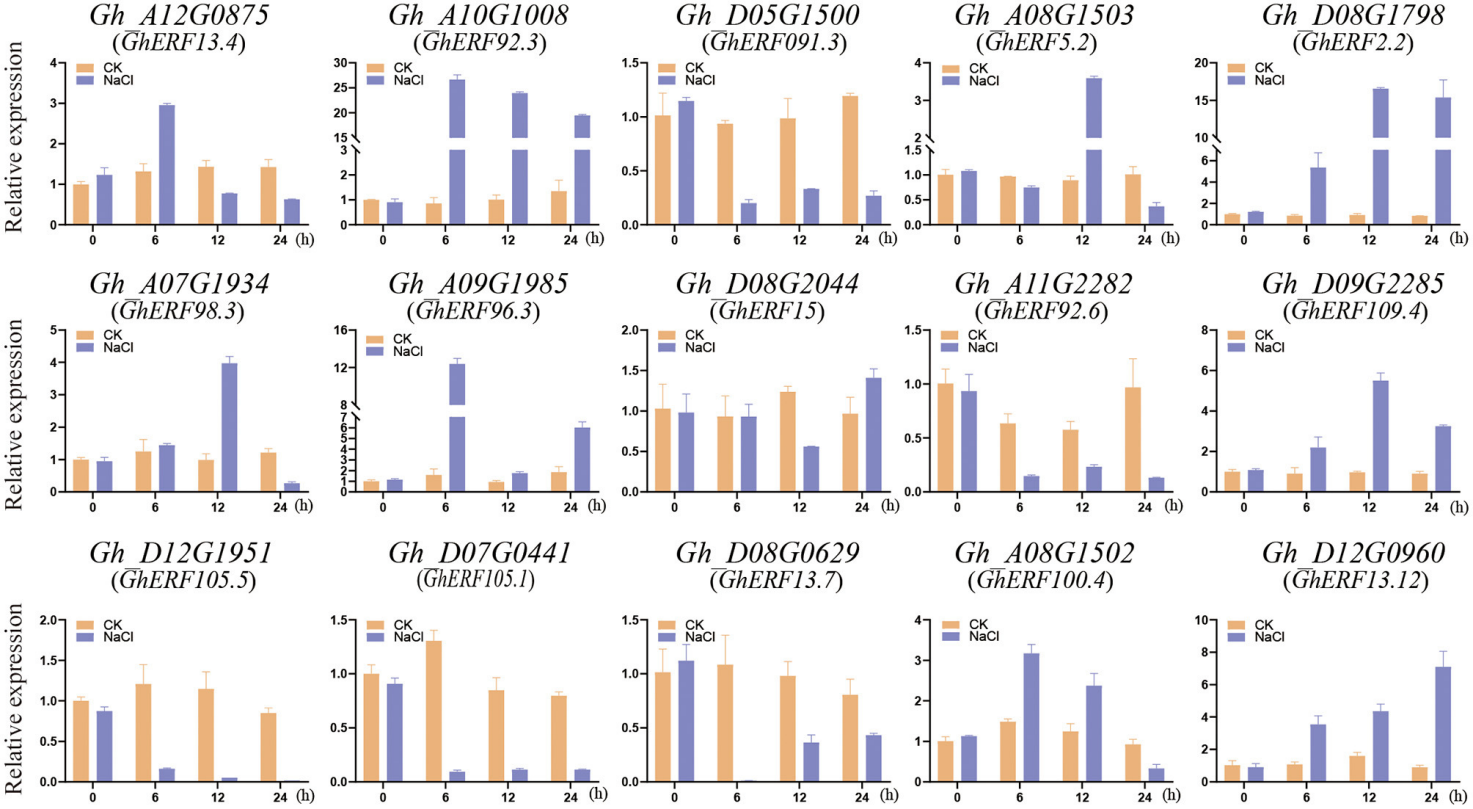
Transcript analysis of the *GhERF* subfamily B3 group genes by salt stress treatment. The response of *G. hirsutum* ERF subfamily B3 group genes to salt stress was examined using real time fluorescent quantitative PCR analysis. The error bars correspond to the standard deviation among three biological replicates.

To understand the functional roles played by the *GhERF* subfamily B3 group genes, we examined the expression patterns of 15 selected *GhERF* subfamily B3 group genes with salt stress and measured transcript levels by qRT-PCR analysis (Figure 3). We evaluated the transcript levels at three time points (6, 12, and 24 h after salt treatment) and compared the results with control CK samples (0 h). After salt treatment, a total of five *GhERF* subfamily B3 group genes were upregulated, namely, *Gh_A10G1008, Gh_D08G1798, Gh_A09G1985, Gh_D09G2285*, and *Gh_D12G0960*. In contrast, the genes *Gh_D05G1500, Gh_A11G2282, Gh_A12G1951, Gh_D07G0441*, and *Gh_D08G0629* were downregulated. Two *GhERF* subfamily B3 group genes (*Gh_A08G1503* and *Gh_A07G1934*) were preferentially expressed at 12 h after the salt treatment, while all other genes showed erratic expression patterns. Interestingly, the genes *Gh_A10G1008* and *Gh_A11G2282*, and *Gh_A12G0875, Gh_D08G0629*, and *Gh_D12G0960* revealed different expression patterns despite being homologous, as they are present in the same sub-branches in the phylogenetic tree (Figure 1).

Among these 15 *GhERF* subfamily B3 group genes, *Gh_D12G0960* was selected for subsequent analysis. The gene contains a full-length CDS region consisting of 630 bp that encodes a putative ERF protein with 209 amino acid residues. Sequence alignment analysis indicated that *Gh_D12G0960* was the closest homolog of the Arabidopsis *AT2G44840.1* (*AtERF13*), with a total of 78.33% sequence identity between both domains. We, thus, termed *Gh_D12G0960* as *GhERF13.12* based on this similarity. *GhERF13.12* has a predicted mass of 23.4 KD, a calculated pI of 4.88, and a protein sequence that includes β-1, β-2, β-3, and α-helix regions with a typical AP2/ERF domain (highlighted with red line) in both *AtERF13* and *GhERF13.12*, as observed in the alignment (Supplementary Figure 3).

**Figure 4:**
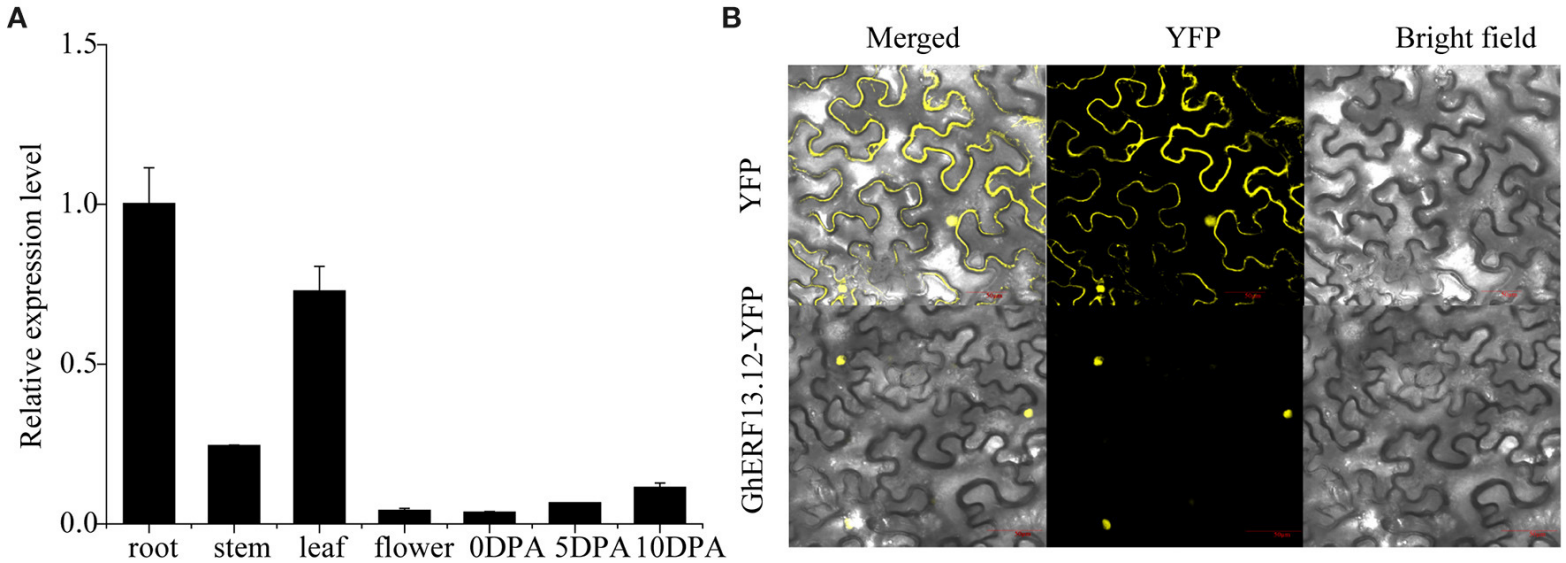
Transcript profile of the *GhERF13.12* gene. **(A)** Tissue-specific expression pattern of *GhERF13.12* obtained by qRT-PCR analysis in the root, stem, leaf, and flower at 0, 5, and 10 DPA (days post-anthesis). Fiber samples were taken from ZM24 cotton plants. The standard deviation among three biological replicates is represented by the error bars. **(B)** Subcellular localization of *GhERF13.12* in tobacco leaves.

The tissue-specific expression pattern of *GhERF13.12* was observed by qRT-PCR in seven vegetative and fiber tissues, such as the root, stem, leaf, and flower and 0, 5, and 10 DPA (days post-anthesis) fiber. The transcript level of *GhERF13.12* was higher in the roots and leaves, which might be related to stress responses (Figure 4A). Moreover, the subcellular localization of GhERF13.12 was determined by transient expression in tobacco leaves, and it was found in the nucleus when compared with the YFP control vector (Figure 4B).

### Ectopic Expression of *GhERF13.12* in Arabidopsis

**Figure 5:**
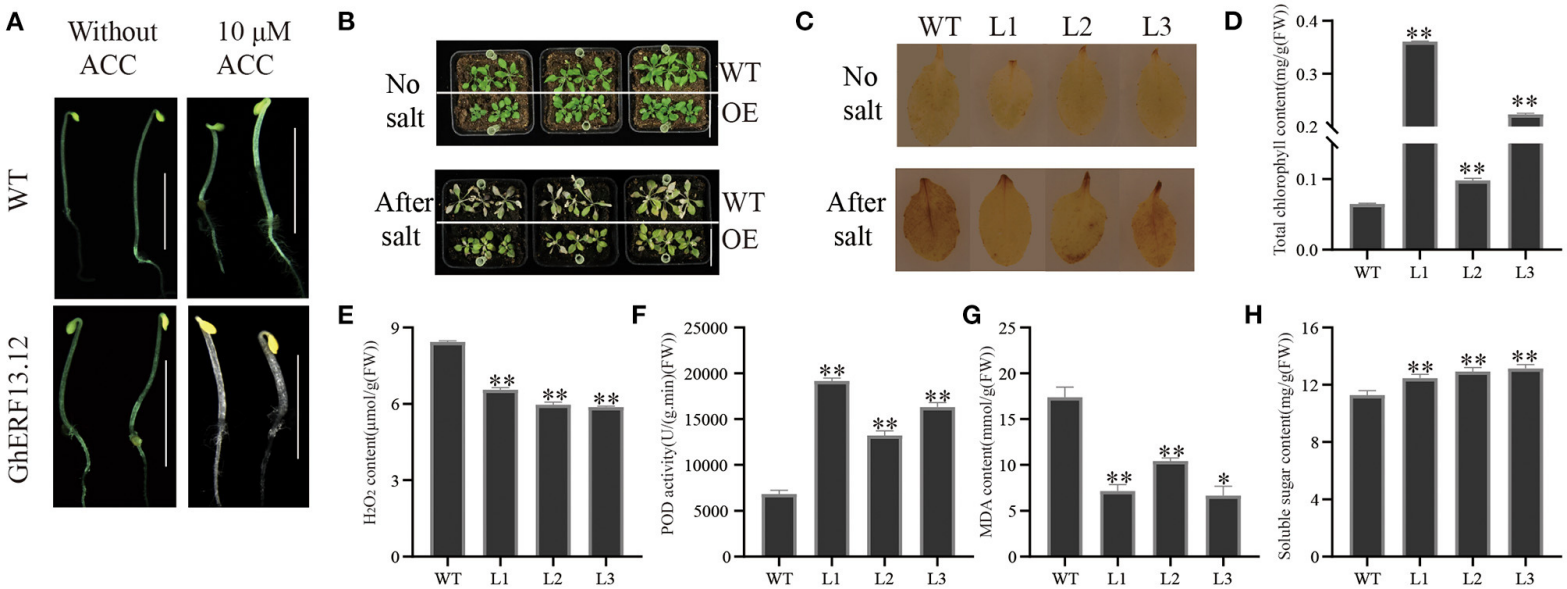
Ectopic expression of the *GhERF13.12* gene in Arabidopsis and salt stress treatment. **(A)** Comparison of hypocotyl length in *GhERF13.12* overexpressing lines and WT plants with and without ACC treatment. **(B)** Salt stress treatment of *GhERF13.12* overexpressing lines and WT plants. Bar = 3 cm. **(C)** DAB staining of *GhERF13.12* overexpressing lines and WT plants with and without salt treatment. Physiological analysis, such as **(D)** total chlorophyll amount, **(E)** H_2_O_2_ content, **(F)** POD activity, **(G)** MDA content, and **(H)** soluble sugar content in WT and *GhERF13.12* overexpressing lines in Arabidopsis. The standard deviation among three biological replicates is shown by the error bars. *p < 0.05 and **p < 0.01 (Student's *t*-test). WT, wild type; L1, L2, and L3, *GhERF13.12* transgenic homozygous lines.

To understand the role of *GhERF13.12*, we overexpressed the coding sequences of the gene in Arabidopsis. The transcript level of *GhERF13.12* in overexpression transgenic Arabidopsis was analyzed by RT-PCR, and we selected three transgenic lines L1–L3 for subsequent experiments (Supplementary Figure 4A). To check the ethylene-evoked triple response in homozygous lines (T3 generation), transgenic seedlings were subjected to ACC treatment (Figure 5A). In general, the results showed that the hypocotyl length in *GhERF13.12* transgenic plants largely decreased by 10 μM following ACC treatment and when compared with WT plants. This indicates that ethylene administered by its precursor ACC inhibited cell elongation and the growth of *GhERF13.12* transgenic seedlings (Figure 5A, Supplementary Figure 4B). These observations are in accordance with previous reports (Wu et al., 2002). To evaluate the function of *GhERF13.12* in response to salt stress, the *GhERF13.12* transgenic plants (L1, L2, and L3) were analyzed with salt treatment. The 20-day-old WT and *GhERF13.12* transgenic plant seedlings were subjected to 250 mM salt treatment for nine consecutive days, and they showed dramatic differences: WT leaves were dry and yellow, while transgenic plant leaves were greener. As an important parameter of plant photosynthetic capacity, we measured chlorophyll content after salt treatment, and found that the *GhERF13.12* overexpressing lines showed higher chlorophyll content compared with WT under stress conditions, indicating these plants are more tolerant to salt than WT plants (Figures 5B,D).

To further elucidate the mechanism of increased salt tolerance in *GhERF13.12* transgenic plants, we performed additional physiological analyses of these transgenic (with salt stress) and WT plants. We found that the amount of MDA and H_2_O_2_ of all the three lines of *GhERF13.12* transgenic plants were lower than WT plants (Figures 5E,G). However, POD activity and soluble sugar content were higher in *GhERF13.12* transgenic plants than WT plants (Figures 5F,H). Furthermore, the plant leaves with and without salt treatment were subjected to DAB staining, and we observed less staining with light color in transgenic plants (Figure 5C). H_2_O_2_ is one of the most important reactive oxygen species and can accumulate in plants with various environmental stresses. The results indicated that the overexpression of *GhERF13.12* reduces ROS-caused damages in Arabidopsis plants under salt stress conditions by reducing H_2_O_2_ content (Li Y. et al., 2019).

**Figure 6:**
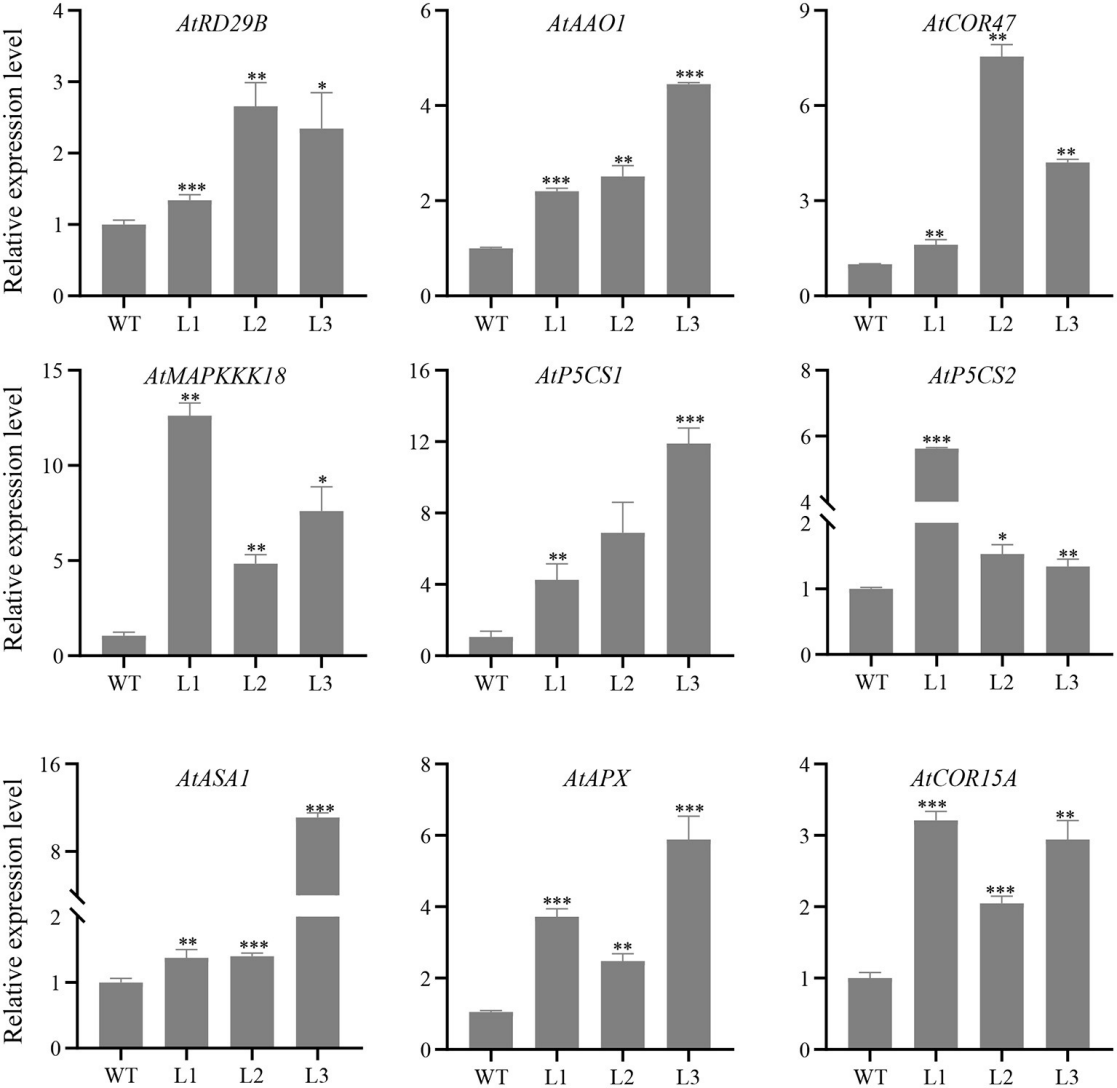
Transcript levels of salt stress-related marker genes. Relative expression patterns of nine genes related to salt stress were examined by qRT-PCR in WT and *GhERF13.12* overexpressing lines. The standard deviation among three biological replicates is shown by the error bars. *p < 0.05, **p < 0.01, and ***p < 0.001 (Student's *t*-test). WT, wild type; L1, L2, and L3, *GhERF13.12* transgenic homozygous lines.

In order to investigate the possible regulatory mechanism of *GhERF13.12* involved in salt stress response, we selected several stress related genes and examined their expression by qRT-PCR in the GhERF13.12-overexpressing and WT Arabidopsis plants with salt stress treatment. We then examined the transcript levels of nine stress-related genes, namely, *AtASA1* (ATP Sulfurylase Arabidopsis 1), *AtAAO1* (abscisic aldehyde oxidase 1), *AtCOR47* (Cold-Regulated 47), *AtMAPKKK18* (MAPKK kinase 18), *AtP5CS1* (pyrroline-5-carboxylate synthetase 1), *AtP5CS2* (pyrroline-5-carboxylate synthetase 2), *AtRD29B* (Responsive to Desiccation 29B), *AtAPX* (Stromal Ascorbate Peroxidase), and *AtCOR15A* (Cold-Regulated 15A) (Figure 6). The qRT-PCR results demonstrated that transcript levels of all the nine genes were relatively higher in all three *GhERF13.12* transgenic lines compared with WT plants under salt stress conditions. Previous studies have shown that these upregulated genes are involved in the ABA signaling pathway and proline biosynthesis pathway, and ROS scavenging (Li Y. et al., 2019). Therefore, we hypothesize that *GhERF13.12* might participate in multiple processes related to salt tolerance.

### Silencing of GhERF13.12 in Cotton

**Figure 7:**
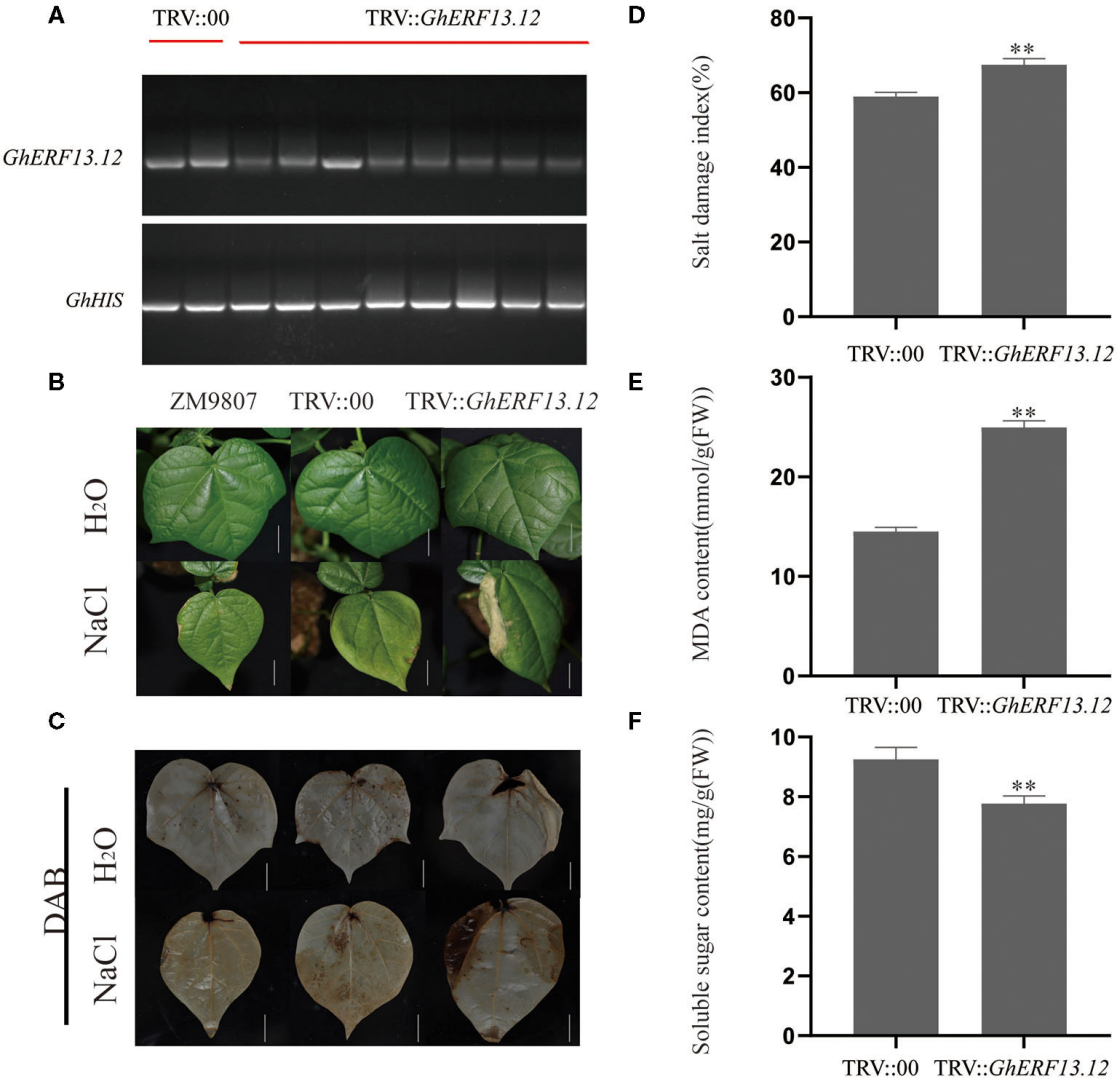
Gene silencing of *GhERF13.12* and salt stress treatment in cotton. *GhERF13.12* was silenced by virus-induced gene silencing (VIGS). **(A)** RT-PCR expression analysis of TRV::00 and TRV::*GhERF13.12* plants to validate the silencing of the *GhERF13.12* gene. *GhHIS3* was used as an internal control. **(B)** Salt stress treatment of ZM9807, TRV::00, and TRV::*GhERF13.12* plants with and without salt stress. Bar = 2.5 cm. **(C)** DAB staining of ZM9807, TRV::00, and TRV::*GhERF13.12* plants with and without salt stress. Other parameters, such as **(D)** salt damage index, **(E)** MDA content, and **(F)** soluble sugar content were observed in TRV::00 and TRV::*GhERF13.12* plants. The standard deviation among three biological replicates is shown by the error bars. **p < 0.01 (Student's *t*-test).

We silenced the *GhERF13.12* gene in cotton plants through a VIGS experiment in order to further validate the functions of this gene. We monitored the efficiency of gene silencing when the leaves of positive control seedlings showed a silencing phenotype (Supplementary Figure 5A) and found that the *GhERF13.12* gene was successfully silenced (Figure 7A). Next, we applied salt stress treatment to WT, control, and *GhERF13.12-*silenced plants and found that the tolerance levels of the *GhERF13.12*-silenced plants to salt stress decreased in comparison with the WT and control plants (Figure 7B). Similarly, soluble sugar content was lower in the *GhERF13.12-*silenced plants than in both the WT and control plants (Figure 7F). Moreover, DAB staining, salt damage index, and MDA content were higher in the *GhERF13.12* silenced plants than in the WT and control plants (Figures 7C–E). The transcript levels of salt stress response genes in the silenced plants were lower than those in the control plants (Supplementary Figure 5B). Collectively, the findings are in accordance with previous studies analyzing the ectopic expression of *GhERF13.12* in Arabidopsis, which validates the authenticity of the findings.

## Discussion

### Evolution of the ERF Subfamily B3 Group Gene Family

Researchers have conducted several studies to clarify the evolutionary history of the ERF TF family in different crop species (Nakano et al., 2006; Jin and Liu, 2008; Zhang et al., 2008; Zhuang et al., 2011; Rashid et al., 2012; Liu et al., 2013; Yan et al., 2013). Here, we report a total of 148 members of the *ERF* subfamily B3 group gene family, including 18 genes in *A. thaliana*, 18 in *G. arboreum*, 94 in *G. hirsutum*, and 18 in *G. raimondii*. On the basis of the Arabidopsis group classification, phylogenetic analysis divided all the cotton ERF subfamily B3 group genes into three groups ranging from A1 to A3, with A3 being the largest group and A2 being the smallest. Phylogenetic classification was similar to previous reports (Ren et al., 2017). Each group showed ERF subfamily B3 group genes from all observed species. Moreover, the *GhERF* subfamily B3 group genes exhibited maximum gene family expansion through massive gene duplication, as evidenced by the fact that *GhERF* subfamily B3 group genes homologous/orthologous gene pairs clustered together in all groups. Accordingly, phylogenetic analysis demonstrated gene duplication was a major factor behind gene family expansion in *G. hirsutum*. Further, sequence analysis showed similar amino acid residue distributions, illustrating that the ERF subfamily B3 group gene family was highly conserved in *G. arboreum, G. hirsutum*, and *G. raimondii* during the evolutionary process. Previously, many genome-wide analyses depicted the expansion and conservation of different gene families in cotton (Ren et al., 2017; Faiza et al., 2019; Qanmber et al., 2019b).

Cotton is an allotetraploid that provides the best model for studying the effects of polyploidy (Yang et al., 2020). The hybridization of the A genome (from *G. herbaceum* and *G. arboreum*) and the D genome (from *G. raimondii*) caused chromosome doubling and the subsequent emergence of nascent At/Dt (allotetraploid cotton) genomes (Li et al., 2015). Here, WGD and segmental duplication represent the main duplication type experienced by the ERF subfamily B3 group gene family in cotton. Gene duplication analysis of the *HH3* and *AAI* gene families in cotton showed they also experienced massive WGD and segmental duplication (Qanmber et al., 2019a,c). Polyploidy contributed to gene duplication events and increased the number of ERF subfamily B3 group gene family members in *G. hirsutum*. Previously, WGD and segmental duplications were found to occur frequently in plants (Cannon et al., 2004), happening twice in Arabidopsis, in the *Brassicaceae* lineage. Cacao and cotton shared a common ancestor and experienced ancient duplication (Li et al., 2014). Gene duplication is a major force of evolution and generates new genes, some of which are redundant (Flagel and Wendel, 2009). We showed that many *GhERF* subfamily B3 group gene pairs were clustered, indicating ancient genome duplication. These pairs experienced rearrangements and shuffling, creating genetic diversity. Previous studies have proposed four possible fates of duplicated genes: (1) the deletion of a gene copy to remove functional redundancy during evolution; (2) sub-functionalization of both copies sharing common functions with parental copies and development of partially different functions through time; (3) one copy acquires a new function (neo-functionalization) during evolution; and (4) maintenance of essential genes for plant growth and development, which is an intermediate between neo- and sub-functionalization processes (Charon et al., 2012).

Collinearity analysis indicated 172 orthologous/paralogous gene pairs between *G. arboreum* and *G. hirsutum*, and 178 orthologous/paralogous gene pairs between *G. hirsutum* and *G. raimondii*. Overall, the *GhERF* subfamily B3 group gene family showed a higher number of orthologous/paralogous gene pairs with *G. raimondii* than with *G. arboreum*. Similarly, 1,392 orthologous/paralogous gene pairs were reported in *G. hirsutum*. These results indicate that the increase in *G. hirsutum* ERF subfamily B3 group genes might result from massive gene duplication due to polyploidy. Previous studies have shown that flowering plants experienced polyploidy events during evolution in order to adapt to changing environmental conditions (Ramsey and Schemske, 1998). This might explain the higher numbers of *G. hirsutum* ERF subfamily B3 group genes and more orthologous/paralogous gene pairs. However, post-hybridization causes gene loss in the process of improved arrangement of genomic sequences together with doubling of chromosomes (Paterson et al., 2004). In contrast, cotton experienced minor changes than Brassica (Gaeta et al., 2007) and paleopolyploid maize (Woodhouse et al., 2010).

Finally, it is often assumed that *Ka/Ks* = 1 indicates neutral evolution, *Ka/Ks* < 1 represents purifying selection, and *Ka/Ks* > 1 suggests positive selection and accelerated evolution (Conant and Wolfe, 2008). The *Ka/Ks* values found between orthologous/paralogous gene pairs in *G. arboreum, G. hirsutum*, and *G. raimondii*, and within *G. hirsutum*, indicated most experienced purifying selection during cotton evolution. Together, the findings provide comprehensive information on polyploidization, chromosomal interaction, and transfer of information *via* inter-genomic hereditary and different rates of evolution, and show that the observed large-scale gene family expansion of ERF subfamily B3 group gene family in *G. hirsutum* was due to segmental or whole genome duplication events.

### Expression Profiles of GhERF Subfamily B3 Group Genes

Several studies elucidated the role of *ERF* genes in response to biotic and abiotic types of stress, especially salt stress (Pandey et al., 2005; Zhang et al., 2007, 2012; Lee et al., 2010, 2015; Zhang L. et al., 2011; Catinot et al., 2015). In this study, some genes were upregulated after salt stress treatment, suggesting the *GhERF* subfamily B3 group genes might play roles in salt stress response. Among these, we noted that *Gh_D12G0960* (*GhERF13.12*) was upregulated at all measured time points. Subsequent analysis confirmed its homology with *AT2G44840.1* (*ERF13*) and showed it contains β-1, β-2, β-3, and α-helix regions and a putative AP2/ERF domain. We further showed that the *GhERF13.12* protein is located in the nucleus.

### *GhERF13.12* Induces Salt Tolerance

ABA is a signaling molecule that takes part in abiotic stress tolerance regulation (such as salt and drought) (Li Y. et al., 2019). *AtERF15* participates in ABA response, and its transgenic lines were sensitive to high salinity and osmotic pressure at the seedling stage (Lee et al., 2015). The transcript levels of two key enzyme genes for ABA biosynthesis increased in overexpressed *JERF1* transgenic rice, which was more drought-tolerant than WT plants (Zhang et al., 2010). *GhERF38* participates in NaCl, mannitol, ABA responses, and reduces plant tolerance to salt stress when overexpressed in Arabidopsis (Ma et al., 2017). In this study, transcripts of *AtAAO1, AtMAPKKK18, AtCOR15A, AtCOR47*, and *AtRD29B*, all of which are involved in abscisic acid-activated signaling pathway, were upregulated in *GhERF13.12* transgenic Arabidopsis under salt stress conditions. Proline is considered a compatible penetrant that can scavenge free radicals, maintain intracellular redox balance, and help maintain the integrity of cell membranes under salt and drought stress conditions, thereby improving salt and drought tolerance (Liu et al., 2014; Zhang et al., 2016; Li Y. et al., 2019; Zhao et al., 2019). *P5CSs* are involved in the proline glutamate biosynthesis pathway, which can increase the accumulation of proline and improve salt and drought tolerance in various plants along with a ROS scavenger that attenuates oxidative stress under high salinity conditions (Yamada et al., 2005; Liu et al., 2014). The transcript of *OsP5CS* increased in *JERF1* transgenic rice under drought stress conditions (Zhang et al., 2010). Here, we showed that the ectopic expression of *GhERF13.12* in Arabidopsis under salt stress conditions and the upregulation of *AtP5CS1* and *AtP5CS2* may lead to proline accumulation. Hence, *GhERF13.12* may regulate gene expression in the ABA signaling and proline synthesis pathways in Arabidopsis under salt stress conditions.

Salt stress induces ionic stress, osmotic stress, and secondary stresses, especially oxidative stress in plants (Yang and Guo, 2018a). Therefore, in order to adapt to salt stress environments, plants rely on signals and pathways to re-establish cellular ion, osmotic, and ROS homeostasis (Yang and Guo, 2018b). White birch *BpERF13* overexpression lines significantly increased tolerance to subfreezing treatment and reduced ROS (Lv et al., 2020). As a non-enzymatic antioxidant, ascorbic acid (AsA) contributes to ROS-scavenging and participates in salt tolerance (Wang and Huang, 2019). AsA biosynthesis enzymes are highly expressed in *AtERF98* overexpressing Arabidopsis, whereby overexpressing plants exhibit salt tolerance (Zhang et al., 2012). Ascorbate peroxidase (APX) is an important enzyme that removes H_2_O_2_ in plants. The overexpression of *PutAPX* in Arabidopsis improves salt stress tolerance by reducing the accumulation of H_2_O_2_ (Guan et al., 2015). It was demonstrated that ATP-sulfurylase contributes to ROS scavenging and maintains reduced cellular-redox environments in plants under salt stress conditions (Anjum et al., 2015). Here, the expression levels of *AtAPX* and *AtASA1* in *GhERF13.12* transgenic plants significantly increased under salt stress conditions (Li Z. et al., 2019). In abiotic stress response, plants can undergo a series of physiological and biochemical changes, in particular in the amount of H_2_O_2_, MDA, and POD activity (Zhu, 2002). The depth of reddish-brown stain formed by the reaction between DAB and endogenous H_2_O_2_ can indicate the level of H_2_O_2_ accumulation. We compared the levels of ROS accumulation in WT and *GhERF13.12* transgenic lines after salinity treatment and found more ROS accumulation in WT (Figure 5C). At the same time, this is consistent with the observed amount of H_2_O_2_ in quantitative analysis, which was lower than that of WT (Figure 5E). POD is a ROS scavenging enzyme that mediates ROS homeostasis in order to protect normal metabolism. A higher POD activity is beneficial to protect the lipid membrane from damage caused by free radicals and to avoid membrane lipid peroxidation caused by unsaturated fatty acids in the membrane (Jiang et al., 2007). We showed POD activity in transgenic plants was higher than that in WT (Figure 5F). Lipid membranes are fragile to stress-induced cellular damage, and the degree of damage is usually used to measure tolerance to the applied stress. MDA is the final product of lipid peroxidation and a reliable indicator of membrane damage caused by stress conditions. In addition, the accumulation of ROS could be insinuated by the MDA content in plant cells (Jain et al., 2001). Here, the accumulation of MDA in the WT plants was higher than in the *GhERF13.12* transgenic lines after salt treatment (Figure 5G). This is in agreement with the lower amounts of MDA accumulated in WT than the *GhERF13.12* silenced plants after salt treatment (Figure 7E). In addition, after salt stress treatment, the transcript levels of salt stress-related genes were lower in the *GhERF13.12* silenced plants than those in the control plants. The results suggest that *GhERF13.12* may act as a stress regulator that increases the scavenging enzyme activity by maintaining ROS homeostasis.

It has been reported that ~6% of land in the world is affected by salt stress, including 22% of arable land and 33% of irrigated croplands used for agricultural practices (Munns and Tester, 2008; Shrivastava and Kumar, 2015; Patel et al., 2020). Cotton is one of the most important natural fiber crops, being used as an edible oil and biofuel, and often faces salinity stress during its lifespan, which reduces fiber production and quality (Longenecker, 1974). Previous genetic engineering studies have shown that ERF genes in cotton, such as *GhERF2*-*GhERF6* (Champion et al., 2009; Jin et al., 2010) and *GhDREB1* (Gao et al., 2009), are involved in the regulation of plant response to salinity. The results provide a meaningful reference for future research on salt tolerance of upland cotton, and show that *GhERF13.12* is an important candidate gene to improve cotton salt tolerance.

## Conclusions

In this study, we identified ERF subfamily B3 group gene family members in *G. arboreum, G. hirsutum*, and *G. raimondii*. Phylogenetic analysis of ERF subfamily B3 group genes in Arabidopsis and three cotton species made it possible to divide these genes into three distinct groups. Cotton ERF subfamily B3 group genes were highly conserved during the course of evolution. The large-scale gene family expansion of the ERF subfamily B3 group gene family in *G. hirsutum* occurred because of segmental or whole genome duplication events, and most genes show typical signatures of purifying selection. At the same time, the *GhERF13.12* sequence is related to constitutive salt stress responses, and the overexpression of *GhERF13.12* transgenic plants have enhanced salt stress tolerance. Furthermore, the overexpression of *GhERF13.12* upregulates transcripts of genes that participate in ABA signaling, proline biosynthesis, and ROS scavenging. These genes lead to increased ABA and proline content, and reduced ROS accumulation, improving tolerance under salt stress conditions. However, the transcript levels of these genes were downregulated in *GhERF13.12* silenced plants after salt treatment. The results of this study will help to clarify the evolution of the ERF B3 subfamily genes in cotton and provide a basis for improving plant tolerance to abiotic stress. In the future, further studies are needed to understand the signal network and the exact function of the *GhERF13.12* gene, along with the molecular mechanism of salt tolerance regulation.

## Data Availability Statement

Publicly available datasets were analyzed in this study. This data can be found at: https://www.cottongen.org/data/download/genome_diploid_A_nd_D5 and https://www.cottongen.org/data/download/genome_tetraploid/AD1#CALL.

## Author Contributions

LLu, GQ, QC, and ZL: conceptualization. GQ, GC, SL, and LLi: software. LLu and GQ: writing—original draft. YW, JL, and MP: formal analysis. MP and WQ: investigation. LLu, MP, and SM: data curation. WQ and SM: methodology. GQ and ZL: writing—review and editing. QC and ZL: supervision. All the authors have read and agreed to the published version of the manuscript.

## Conflict of Interest

The authors declare that the research was conducted in the absence of any commercial or financial relationships that could be construed as a potential conflict of interest.

## Publisher's Note

All claims expressed in this article are solely those of the authors and do not necessarily represent those of their affiliated organizations, or those of the publisher, the editors and the reviewers. Any product that may be evaluated in this article, or claim that may be made by its manufacturer, is not guaranteed or endorsed by the publisher.

## Supplementary Material

Supplementary Figure 1 Conserved amino acid sequence in three cotton species, such as *G. arboreum, G. raimondii*, and *G. hirsutum*.

Supplementary Figure 2 Collinearity analysis of the *GhERF* subfamily B3 group genes. Collinearity analysis of *G. hirsutum* ERF subfamily B3 group genes among and within the A- and D-subgenomes is represented by the red lines.

Supplementary Figure 3 Sequence alignment among ERF13 proteins. Sequence alignment of *A. thaliana* and *G. hirsutum* ERF13 proteins depicted β-1, β-2, β-3, and α-helix regions, and a putative AP2/ERF domain (red region).

Supplementary Figure 4 Transcript levels and hypocotyl length in *GhERF13.12* transgenic Arabidopsis. **(A)** Transcript levels of *GhERF13.12* in transgenic Arabidopsis as measured by RT-PCR. **(B)** Determination of hypocotyl length in *GhERF13.12* transgenic Arabidopsis with or without ACC in MS.

Supplementary Figure 5 Phenotype of virus-induced gene silencing (VIGS) plants and transcript levels of salt stress related genes. **(A)** Silenced phenotype of positive control seedlings during VIGS experiment. **(B)** Transcript levels of salt stress-related genes in *GhERF13.12*-silenced plants with salt stress.

Supplementary Table 1 List of primers used in this study (5′-3′).

Supplementary Table 2 Gene IDs of all identified ERF subfamily B3 group genes in *A. thaliana, G. arboreum, G. raimondii*, and *G. hirsutum*.

Supplementary Table 3 Type of gene duplication in *G. arboreum, G. raimondii* and *G. hirsutum* ERF subfamily B3 group genes.

Supplementary Table 4 Cotton ethylene response factor (ERF) subfamily B3 group genes orthologous and paralogous gene pairs and their respective Ka/Ks values in *G. arboreum, G. raimondii*, and *G. hirsutum*.

Supplementary Table 5 *G. hirsutum* ethylene response factor (ERF) subfamily B3 group genes orthologous and paralogous gene pairs and their respective Ka/Ks values.

## Abbreviations

ERF: Ethylene Response Factor
WGD: Whole Genome Duplication
MDA: Malondialdehyde
POD: Peroxidase
ROS: Reactive Oxygen Species
NAU: Nanjing Agricultural University
JGI: Joint Genome Institute
ICR: Institute of Cotton Research.
