# Supplementary figures and images for: Identification and Characterization of the ERF Subfamily B3 Group Revealed GhERF13.12 Improves Salt Tolerance in Upland Cotton
Source: Front Plant Sci. 2021 Aug 9;12:705883. doi: 10.3389/fpls.2021.705883 (PMC8382128; doi:10.3389/fpls.2021.705883)

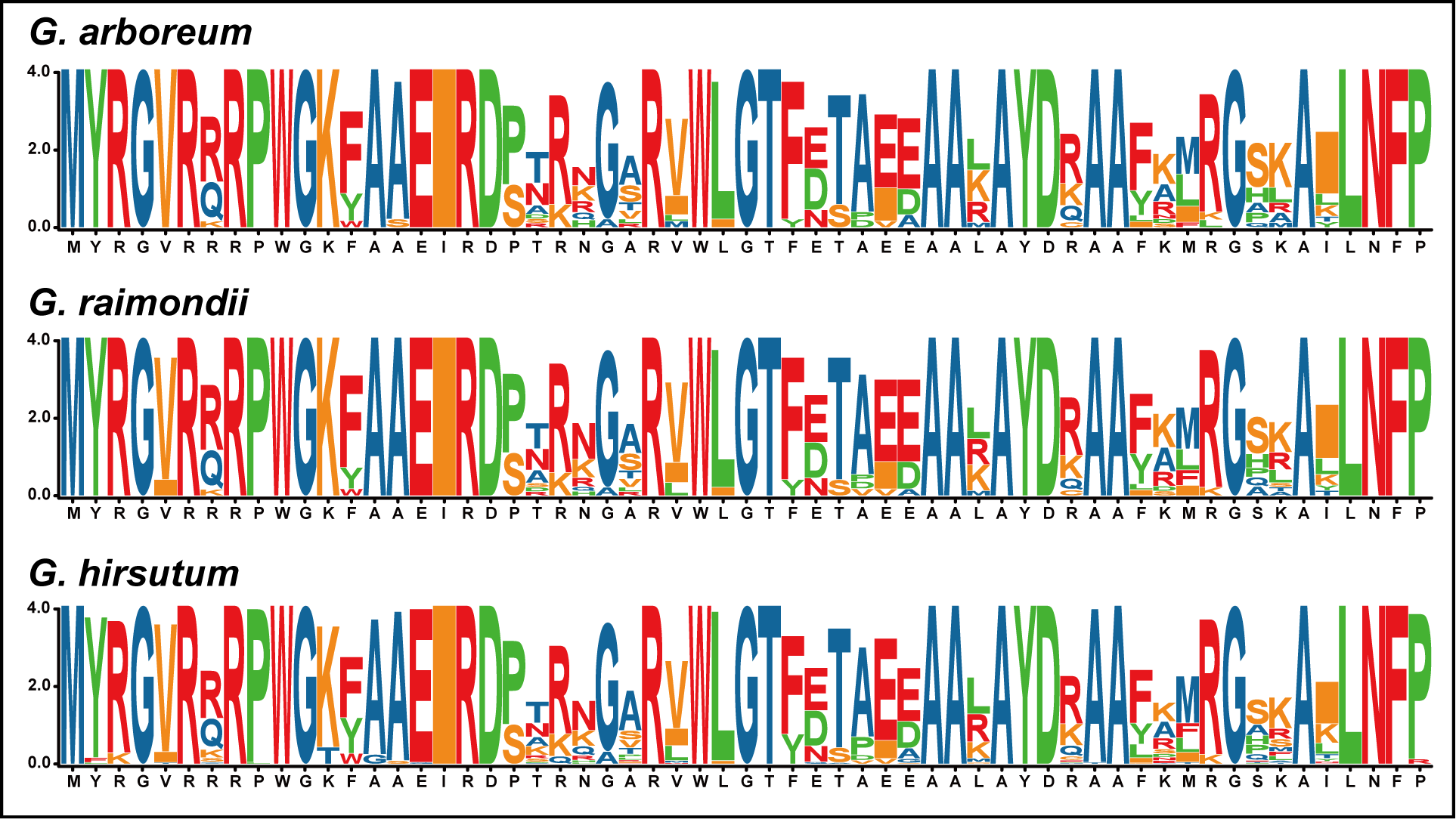

Supplement: Supplementary Figure 1 — Conserved amino acid sequence in three cotton species, such as G. arboreum, G. raimondii, and G. hirsutum. [file Image_1.TIF]

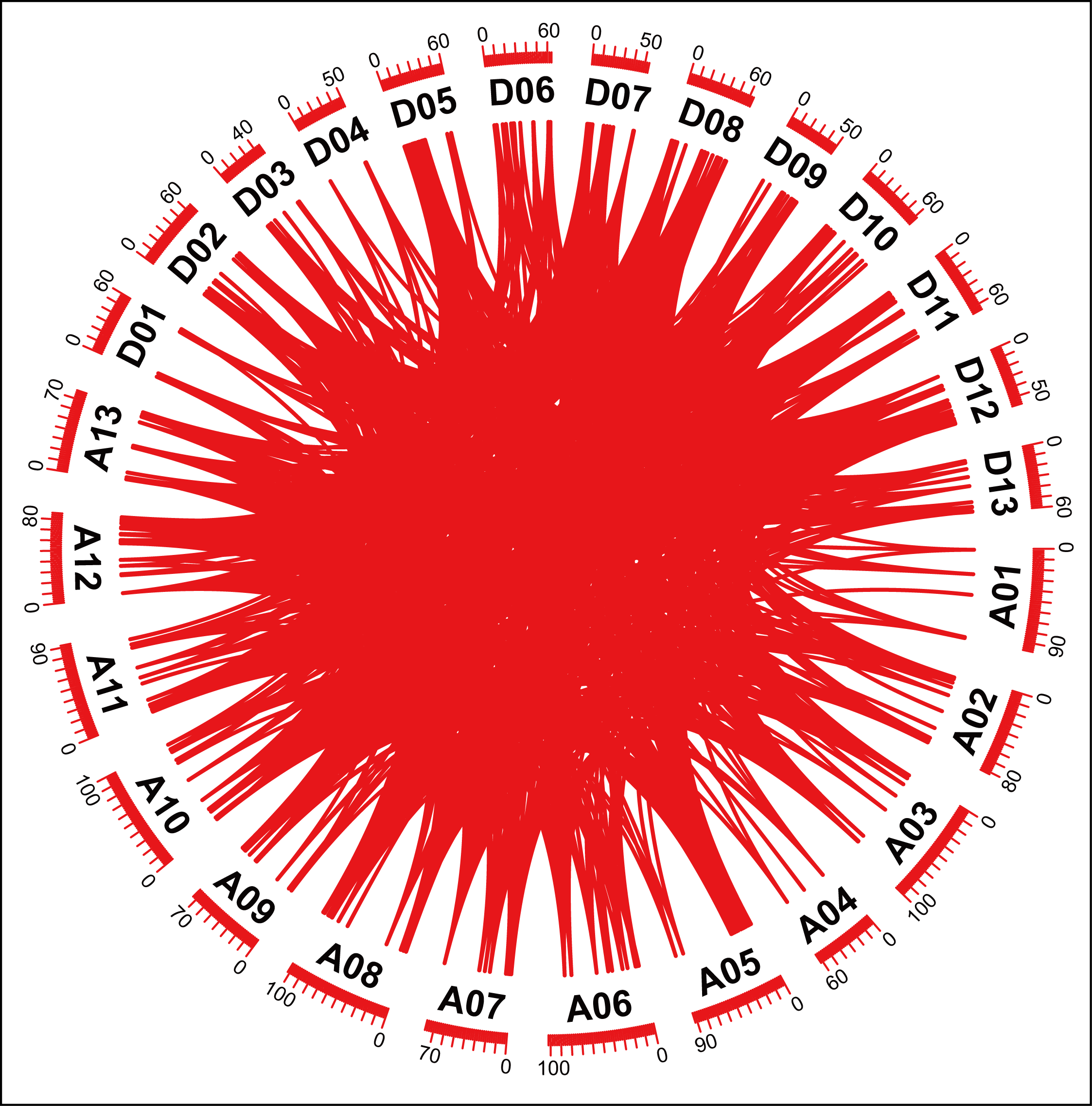

Supplement: Supplementary Figure 2 — Collinearity analysis of the GhERF subfamily B3 group genes. Collinearity analysis of G. hirsutum ERF subfamily B3 group genes among and within the A- and D-subgenomes is represented by the red lines. [file Image_2.TIF]

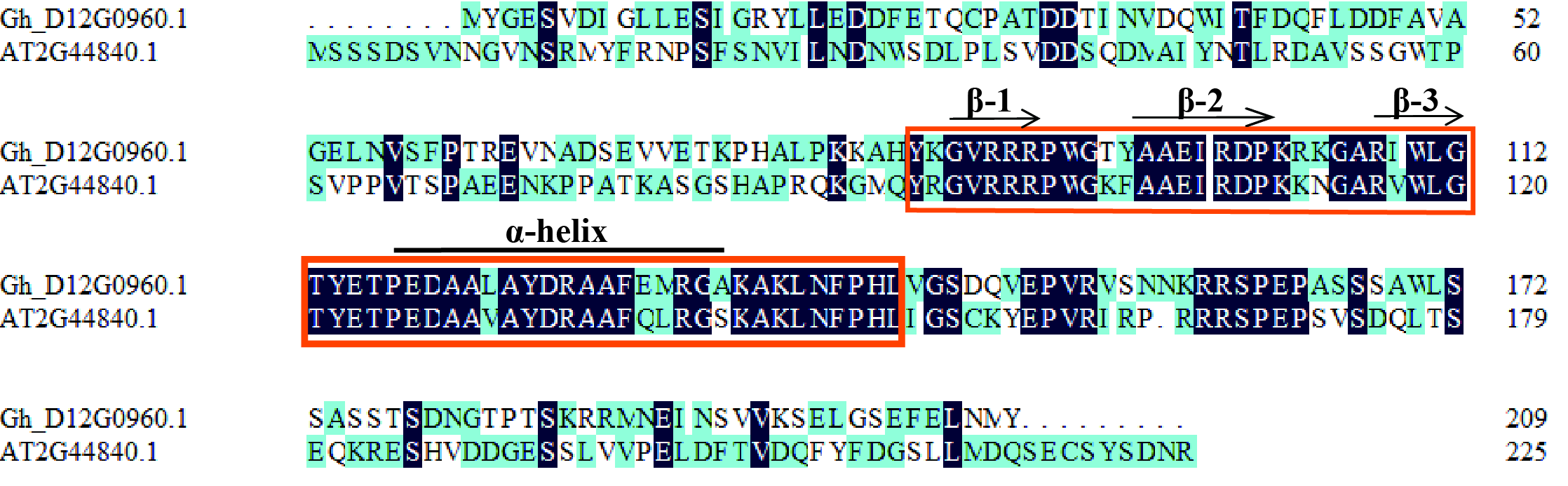

Supplement: Supplementary Figure 3 — Sequence alignment among ERF13 proteins. Sequence alignment of A. thaliana and G. hirsutum ERF13 proteins depicted β-1, β-2, β-3, and α-helix regions, and a putative AP2/ERF domain (red region). [file Image_3.TIF]

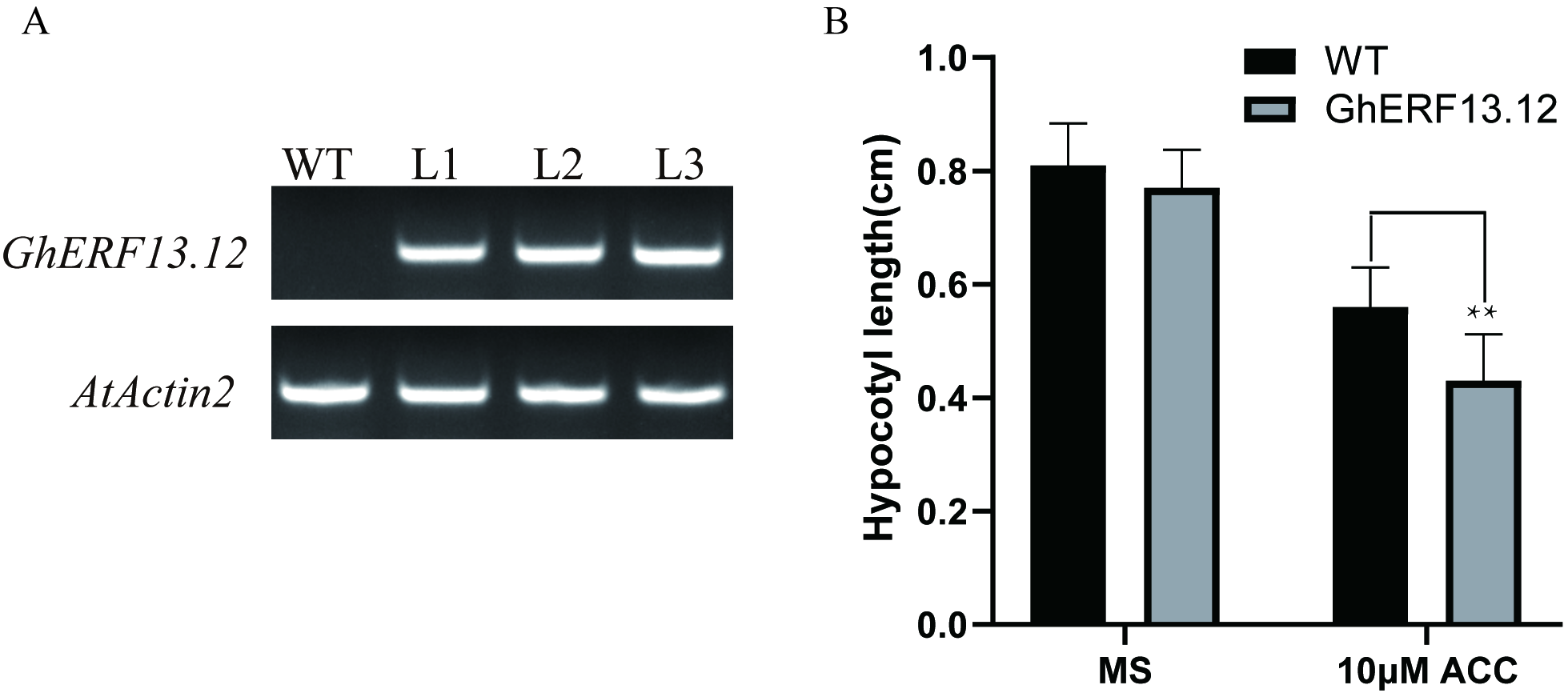

Supplement: Supplementary Figure 4 — Transcript levels and hypocotyl length in GhERF13.12 transgenic Arabidopsis. (A) Transcript levels of GhERF13.12 in transgenic Arabidopsis as measured by RT-PCR. (B) Determination of hypocotyl length in GhERF13.12 transgenic Arabidopsis with or without ACC in MS. [file Image_4.TIF]

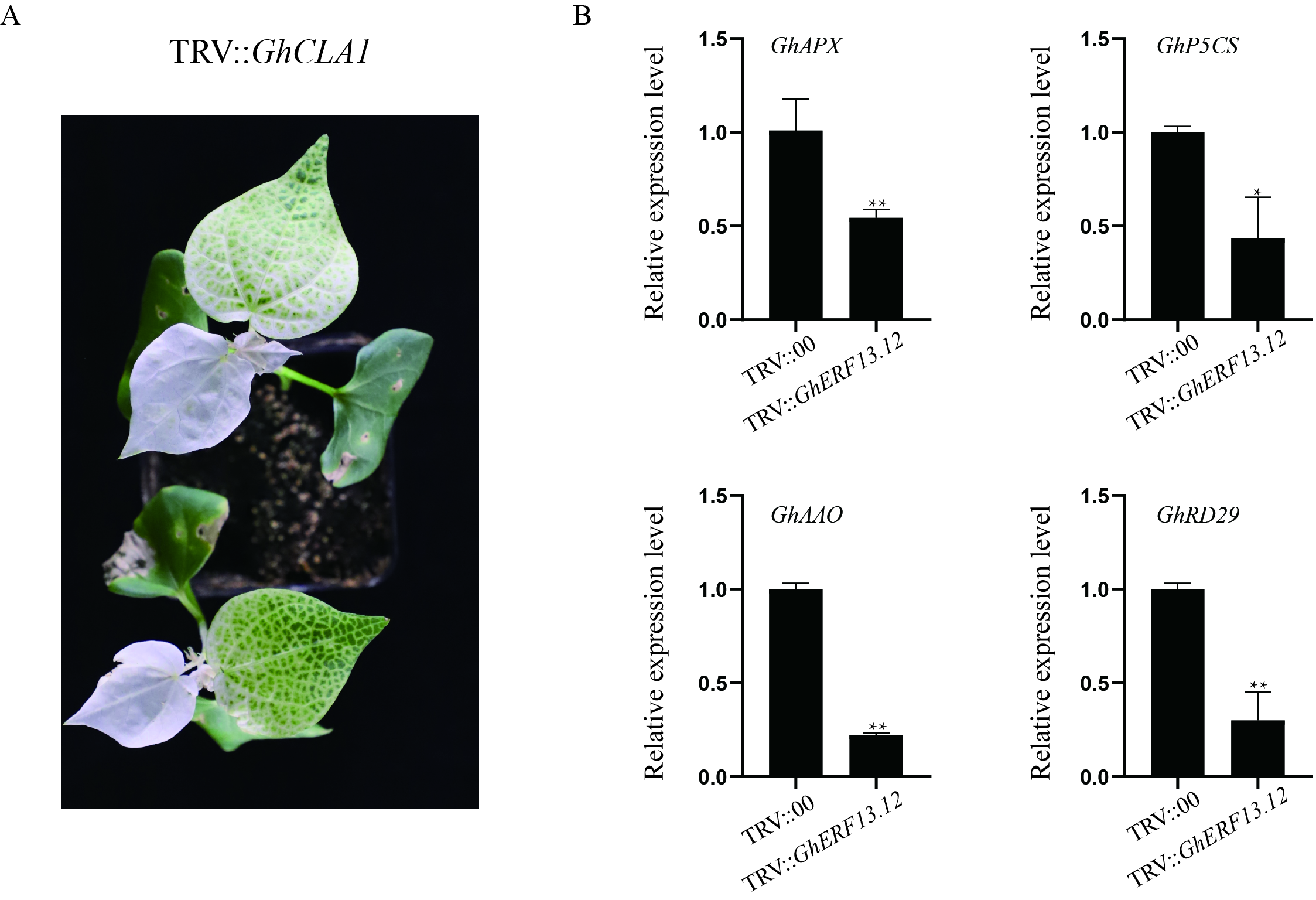

Supplement: Supplementary Figure 5 — Phenotype of virus-induced gene silencing (VIGS) plants and transcript levels of salt stress related genes. (A) Silenced phenotype of positive control seedlings during VIGS experiment. (B) Transcript levels of salt stress-related genes in GhERF13.12-silenced plants with salt stress. [file Image_5.TIF]
